# BAC libraries construction from the ancestral diploid genomes of the allotetraploid cultivated peanut

**DOI:** 10.1186/1471-2229-8-14

**Published:** 2008-01-29

**Authors:** Patricia M Guimarães, Olivier Garsmeur, Karina Proite, Soraya CM Leal-Bertioli, Guilhermo Seijo, Christian Chaine, David J Bertioli, Angelique D'Hont

**Affiliations:** 1 Biotechnology Unit, Embrapa Genetic Resources and Biotechnology, Brasília, DF, Brazil; 2 Centre de Coopération International en Recherche Agronomique pour le Developpement (CIRAD), Montpellier, France; 3 Cell Biology Department, IB-University of Brasília (UnB), Brasília, DF, Brazil; 4 Plant Cytogenetics and Evolution Laboratory, Instituto de Botánica del Nordeste, Corrientes, Argentina; 5 Biotechnology and Genomic Sciences Department, Campus II Catholic University of Brasília, Brasília, DF, Brazil

## Abstract

**Background:**

Cultivated peanut, *Arachis hypogaea *is an allotetraploid of recent origin, with an AABB genome. In common with many other polyploids, it seems that a severe genetic bottle-neck was imposed at the species origin, via hybridisation of two wild species and spontaneous chromosome duplication. Therefore, the study of the genome of peanut is hampered both by the crop's low genetic diversity and its polyploidy. In contrast to cultivated peanut, most wild *Arachis *species are diploid with high genetic diversity. The study of diploid *Arachis *genomes is therefore attractive, both to simplify the construction of genetic and physical maps, and for the isolation and characterization of wild alleles. The most probable wild ancestors of cultivated peanut are *A. duranensis *and *A. ipaënsis *with genome types AA and BB respectively.

**Results:**

We constructed and characterized two large-insert libraries in Bacterial Artificial Chromosome (BAC) vector, one for each of the diploid ancestral species. The libraries (AA and BB) are respectively *c*. 7.4 and *c*. 5.3 genome equivalents with low organelle contamination and average insert sizes of 110 and 100 kb. Both libraries were used for the isolation of clones containing genetically mapped legume anchor markers (single copy genes), and resistance gene analogues.

**Conclusion:**

These diploid BAC libraries are important tools for the isolation of wild alleles conferring resistances to biotic stresses, comparisons of orthologous regions of the AA and BB genomes with each other and with other legume species, and will facilitate the construction of a physical map.

## Background

Cultivated peanut (*Arachis hypogaea *L) is the second-most important grain legume crop worldwide after soybean, with a production of 33 million tons in 2003/04 [1]. Peanut is produced throughout the tropics and warmer regions of the subtropics, but is particularly important in Africa, Asia and in the United States [1]. It is an allotetraploid (AABB) of recent origin that arose from hybridization of two wild species and spontaneous chromosome duplication [2,3]. This polyploidization event gave rise to a severe genetic bottle-neck [2,3] which has led to lack of variability in some important traits, limited availability of allelic combinations and consequently restrictions in productivity. In addition, the very low level of polymorphism in cultivated peanut has hampered genetic and genomic characterization. In contrast, diploid wild relatives of peanut have high genetic diversity and have been selected during evolution in a range of environments and biotic stresses, constituting a rich source of allele diversity [3]. Wild alleles can be introduced into the gene-pool of cultivated peanut using a "resynthesis" pathway, essentially artificially recreating events similar to those that gave rise to the origin of the crop species [4]. Recent advances in the knowledge of the relationships of wild and cultivated genomes through traditional taxonomy, cytogenetics and molecular studies are enabling new choices of wild species for resynthesis [5-8]. In parallel new genetic and genomic tools (see below) for monitoring the introgression of wild genes into a cultivated background are opening the perspectives for the efficient introgression of wild genes into the peanut crop using molecular breeding.

The very low level of polymorphism in cultivated peanut has hampered genetic mapping and QTL (Quantitative trait loci) studies. Consequently only a few linkage maps have been published. All of them have used wild species to enable the generation of sufficient polymorphic markers. Restriction fragment length polymorphism (RFLP) maps were developed by Halward based on a cross of two AA genome species, *A. stenosperma *Krapov. & WC Gregory and *A. cardenasii *Krapov. & WC Gregory, and a tetraploid map based on a cross of TxAG-6 (a synthetic amphiploid) and *A. hypogaea *was published by Burow et al. [9]. Recently, we developed an SSR-based map for the AA genome of *Arachis *based on a cross of *A. stenosperma *and *A. duranensis *Krapov. & WC Gregory [10] and a map of the BB genome, based on a cross of *A. ipaënsis *Krapov., WC Gregory & CE Simpson and *A. magna *Krapov., WC Gregory & CE Simpson [11]. Currently there are 54,168 ESTs for *A. hypogaea *in Genbank [12-14], and 6,264 for the wild AA genome *A. stenosperma *[15,16].

Bacterial Artificial Chromosome (BAC) libraries are fundamental tools for genomic studies, being important for physical mapping, map-based gene cloning and analysis of gene structure and function. The easy handling and propagation of the clones, their relatively stability and low degree of chimerism compared with yeast artificial chromosome (YAC) vectors have made BAC vectors the cloning system of choice [17,18]. A number of strategies have been proposed for physical mapping with large-insert clones: hybridisation-based methods such as interactive hybridisation using individual cDNA or genomic clones as probes [19], restriction-based fingerprinting methods [20] integrated BAC end sequencing and fingerprint analysis [21] or more recently, the use of oligonucleotide-based "overgos" [22].

Within the Leguminosae, BAC libraries are available for *Phaseolus vulgaris *[23], *Vigna radiata *[24], *Glycine max *[25], *Trifolium pretense *[26] and the model legumes *Lotus japonicus *[27] and *Medicago truncatula *[28]. Within the genus *Arachis*, one BAC library for the allopolyploid cultivated peanut has been developed [29]. As a complement to this resource, here we describe the production of BAC libraries for the two diploid wild species *A. duranensis *(AA genome) and *A. ipaënsis *(BB genome) that have been identified as the most probable ancestors of cultivated peanut [8,30]. Using whole genome *in-situ *hybridization (GISH) we also further investigated the affinities and coverage of the selected diploid genomes compared to those present in *A. hypogaea*.

## Results

### *In-situ *hybridizations

**Figure 1:**
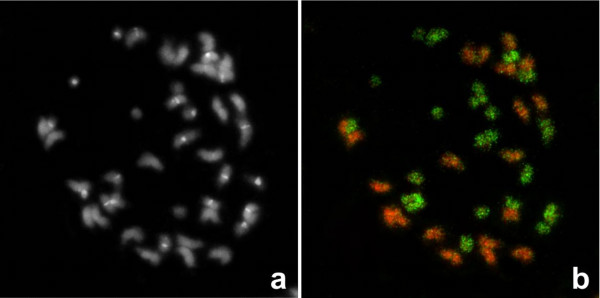
**GISH of *Arachis hypogaea *metaphase chromosomes**. Somatic metaphases of *Arachis hypogaea *(subsp. *hypogaea *var. *hypogaea*, race Guaycurú) after a) 4'6-diamidino-2-phenylindole (DAPI) counterstaining (blue, shown in black and white), b) Genomic *in situ *hybridization using genomic DNA from *A*.*duranensis *(in green) and *A. ipaënsis *(red).

Total genomic DNA of *A. duranensis *(AA genome) and *A. ipaënsis *(BB genome) when used as probes on the chromosomes of *A. hypogaea *displayed intense and uniform hybridization patterns onto AA and BB chromosomes of *A. hypogaea *respectively (Fig. 1). This clear genome discrimination of the chromosome subsets from the corresponding parental genomes in the tetraploids was possible without the need of any unlabelled blocking DNA, which is normally used to avoid cross-hybridization between a specific probe from one genome and homologous DNA sequences from another genome. Counterstained A and B chromosomes show very similar total sizes for the two genomic components (Fig. 1).

### High Molecular Weight (HMW) DNA isolation

The use of standard HMW DNA isolation protocols [31,32] did not produce sufficient amounts of good quality nuclei for *Arachis*. High levels of carbohydrate and polyphenols are present in both *A. duranensis *and *A. ipaënsis *leaves. This results in high viscosity leaf extracts, which are difficult to filtrate, increasing considerably the time of the nuclei exposure to the action of oxidizing substances. To overcome this problem, some modifications were necessary: inclusion of PVP-40 in the extraction buffer, filtration of leaf extracts in four layers of cheesecloth followed by two layers of Miracloth, centrifugation at low speed (60 × g for 2 min) and a Percoll gradient (37.5%). The analysis of the extracts by DAPI-staining microscopy enabled the correct evaluation of the amount and quality of the nuclei preparations.

The inclusion of an extra purification step, consisting of PFGE of agarose plugs for 40 min before digestion, enabled the purging of smaller fragments and eliminated impurities, increasing cloning efficiency. To obtain the highest amount of restricted DNA after electro-elution, only 50 μL was recovered at the very bottom of the collection tube instead of 300 μL as described in the standard protocol [21]. After the double size selection, various ratios of ligation were tested with the 1/4 V/I ratio resulting in the greatest number of transformants. Overall, five different ligations were necessary to obtain each BAC library (Table 1).

**Table 1:** Characteristics of the *A. duranensis *and *A. ipaënsis *BAC libraries

**Species**	**Ligations**(no.)	**Genome size **(Mb)	**Clones **(no.)	**Average insert size **(kb)	**mtDNA**(%)	**cpDNA**(%)	**Estimated genome coverage**
*A. duranensis*	5	1260^a^	84,096	110	0.21	0.016	7.4×
*A. ipaënsis*	5	1415–2830^b^	75,648	100	0.081	0.363	5.3–2.7^b^x

mt: mitochondrial, cp: chloroplastic

^a ^Temsch & Greilhuber, 2001

^b ^Singh *et al*, 1996

### BAC libraries characterisation

**Figure 2:**
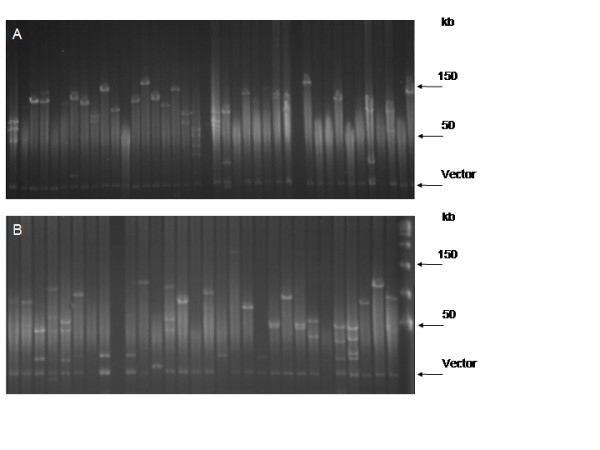
***A. duranensis *(a) and *A. ipaënsis *(b) libraries sizing**. Random BAC clones from the *A. duranensis *(a) and *A. ipaënsis *(b) libraries digested with *No*tI and separated by PGEF. The size of a few reference bands from Lambda Ladder PFG Marker (New England Biolabs) are indicated in kilobases.

**Figure 3:**
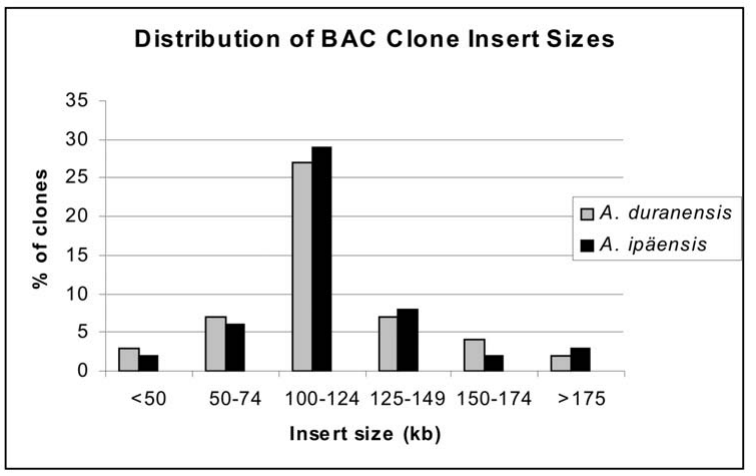
**BAC libraries insert sizes**. Distribution of insert sizes from randomly selected BAC clones from the *A. duranensis *and *A. ipaënsis *libraries.

The BAC library for the AA genome (*A. duranensis*) contained 84,096 clones whilst the BB genome library (*A. ipaënsis*) consisted of 75,648 (Table 1). A random sample of each library was analyzed by *Not*I digestion, and the average insert size was 110 and 100 kb for *A. duranensis *and *A. ipaënsis *respectively (Fig. 2a and 2b; Fig. 3).

The organelle contamination in both BAC libraries was evaluated by screening the high-density filters with mitochondrial and chloroplast specific probes. For *A. duranensis*, the contamination of BAC clones with chloroplast sequences was of 0.016% and mitochondrial sequences of 0.21%. For *A. ipaënsis *0.363% was contaminated with chloroplast sequences and 0.081% of the clones with mitochondrial DNA. These values, together with the microscopic DAPI-staining observations, reflect the high level of purification of the *Arachis *nuclei obtained with the modified protocol.

Based on the library average insert size and *A. duranensis *haploid genome, equivalent to 1260 Mb [33], the estimated coverage of the AA genome BAC library is of 7.4 haploid genome equivalents. However, for *A. ipaënsis*, the DNA-content determination is controversial. It is possible that the haploid genome equivalent of 2,830 Mb reported by Singh et al [34] is a 2.0 fold overestimate because of measurement inconsistencies, as already described for other *Arachis *species [33,35] Therefore the BB genome BAC library for *A. ipaënsis *could represent from 2.7 to 5.3 the haploid genome equivalents of the species. Considering that A and B chromosomes show very similar sizes for the two genomic components of the tetraploid (as mentioned above, Fig. 1) we consider that latter to be a better estimate. To further test the coverage, high-density filters were screened with probes corresponding to single copy gene used as anchor markers in legume and that have been placed on the *Arachis *AA genetic map (unpublished data). An average of 5.1 clones per probe was identified in the AA genome and 4.5 in the BB (Table 2).

**Table 2:** BAC library filter-hybridization results using legume single-copy probes and one RGA

**Probe**	**Number of BAC clones detected**	
	***A. duranensis***	***A. ipaënsis***

RGA S1_A_36	2	0
Leg083	2	5
Leg128	4	4
Leg092, Leg149, Leg178 (mixed)	19	
Leg 92	10	*
Leg237	5	*
Leg242	6	*
Leg 88	0	*
Average Leg clones	5,11	4,5

* Not tested

The hybridization of both BAC libraries with an *Arachis *resistance gene analogue RGA S1_A_36 identified two clones in the AA genome but none in the BB genome (Table 2).

## Discussion

Cultivated peanut is an allotetraploid with two nuclear genomic components, AA and BB. Although it is generally agreed that these component genomes are derived from diploid wild ancestors, the exact species involved has been a matter of some research and discussion. Although the evidence is not completely clear cut, analysis of data from molecular markers, cytogenetics, morphology and geographical distributions support that *A. duranensis *and *A. ipaënsis *are the direct ancestors of cultivated peanut [8,30].

Genomic *in situ *hybridization (GISH) of *A. hypogaea *metaphase chromosomes with total genomic DNA from the AA genome of *A. duranensis *and the BB genome of *A. ipaënsis *allowed a clear differentiation of the A and B chromosomes. Firstly, this observation reinforces the evidence of the close relationship between the genomes of *A. duranensis, A. ipaënsis *and cultivated peanut. Secondly, since GISH relies largely on the hybridization of repetitive sequences, it also indicates that *A. duranensis *and *A. ipaënsis *genomes have diverged substantially regarding their repeated sequences/transposable elements contents.

In contrast, the evidence available indicates that the gene order in the AA and BB genomes is substantially conserved [9]. This situation of largely syntenic gene frameworks embedded within quickly evolving repetitive DNA seems to be a recurrent theme in plant evolution [36-39].

The availability of BAC libraries from the allopolyploid and the two wild ancestors will allow the comparison of these genomes regarding microsynteny and repetitive DNA contents, in particular transposable elements and will provide insights into the fascinating area of polyploid genome evolution.

In addition, the legume single copy anchor markers, used to characterize the libraries, will allow orthologous regions to be identified and compared between the AA and BB *Arachis *genomes, and with other legume genomes, in particular the sequenced genomes of *Lotus *and *Medicago*, providing insight into legume genome evolution.

Construction of BAC libraries in some plants can require substantial effort due to particular chemical constitution as already experienced by us before [32]. For *Arachis*, obtaining good quality HMW DNA was difficult due to contamination from polyphenols and carbohydrates, which are abundant in *Arachis *leaves. A number of steps had to be added to the standard protocols of nuclei extraction and several ligations had to be made to obtain a reasonable number of BAC clones. These difficulties were also reported by Yuksel and Paterson [29] for the construction of the *A hypogaea *BAC library.

The genome coverage of the BAC libraries was estimated between 7.4 and 5.1 haploid genome equivalents for the A genome and 5.3 to 4.5 for the B genome. These disparities are due to variations of the density of restriction sites in certain genome regions or difficulties in cloning too large or too small fragments [40]. Considering that 99% coverage is equivalent to 4.7× haploid genomes [40]*A. duranensis *and *A ipaënsis *genomes are well represented and the libraries will be suitable for many applications.

The availability of BAC libraries from diploid *Arachis *will greatly facilitate the development of a reference physical map for *Arachis*. The construction of this physical map will be initiated soon with the *A. duranensis *BAC library. This species was used as a parent of the mapping population used for the construction of the *Arachis *SSR-based map [10]. *A. ipaënsis *is also the parent of a mapping population [11]. This will facilitate the integration of genetic and physical maps.

The exploitation of peanut's diploid wild relatives for breeding is very attractive since they possess various resistances to biotic and abiotic stresses. The two species used to make the BAC libraries harbour resistances to nematode and fungal diseases[41,42]. These new BAC resources will help the tagging and/or the isolation of the corresponding resistance genes. For example, a QTL for resistance to late leaf spot, caused by *Cercosporidium personatum *has been mapped in a cross of *A. duranensis *with *A. stenosperma *[43]. S1_A_36, a RGA that co-segregates with this QTL has been used to identify two BAC clones in the *A. duranensis *library, whilst no clones in the *A. ipaënsis *library were found. Sequencing of the BAC clones should enable the identification of microsatellites in the target region, thus providing more convenient markers for tracking the QTL in segregating populations.

## Conclusion

In summary, here we describe the production of BAC libraries for the AA and BB genomes of *Arachis*. The libraries will be a useful resource for the isolation of genes, the construction and correlation of physical and genetic maps, the isolation of probes for cytogenetic analysis, the study of the evolution of the two genome types, and, by comparison with the allotetraploid genome of cultivated peanut, for the study of the evolution of polyploid genomes.

## Methods

### Plant Material

Seeds were obtained from the *Arachis *germplasm collection, maintained at Embrapa Genetic Resources and Biotechnology – CENARGEN (Brasília-DF, Brazil). The germination was improved by placing the seeds on a blotting paper humidified with a 1% Ethephon. For HMW DNA isolation, *A. duranensis *V14167 (genome AA) and *A. ipaënsis *KG30076 (genome BB) were grown under greenhouse conditions. Young leaves were collected in liquid nitrogen then stored at -80°C.

### Probe labelling and fluorescent *in situ *hybridization

Whole genomic DNA from *A. duranensis *and *A*. *ipaënsis *were used as probes in genomic *in situ *hybridization (GISH). Probes were labelled with digoxigenin-11-dUTP (Roche, Mannheim, Germany) or biotin-11-dUTP (Sigma) by nick translation.

Pre-treatment of preparations, chromosome and probe denaturation, conditions for the *in situ *hybridization (hybridization mixes contained DNA probes at a concentration of 2.5–3.5 ng/μL), post-hybridization washings, blocking, and indirect detection by fluorochrome conjugated antibodies were performed according to Moscone et al[44]. Antibodies consisted of mouse anti-biotin (Dakopatts, Dako, Carpinteria, California, USA) and sheep anti-digoxigenin conjugated to fluorescein isothiocyanate (FITC) (Roche) in PBS (0.13 mol/L NaCl, 0.007 mol/L Na_2_HPO_4_, 0.003 mol/L NaH_2_PO_4_), 3% (w/v) bovine serum albumin (BSA) or, rabbit anti-mouse conjugated to tetramethyl-rodamine isothiocyanate (TRITC) (Dakopatts) and FITC-conjugated rabbit anti-sheep (Dakopatts) in PBS, 3% (w/v) BSA. Preparations were counterstained and mounted with Vectashield medium (Vector Laboratories, Burlingame, California, USA) containing 2 μg/mL of 4',6-diamidino-2-phenylindole (DAPI, Sigma).

The DAPI counterstaining subsequent to GISH resulted in a C banding-like pattern with major heterochromatin bands fluorescing more intensely, thus aiding chromosome identification [8,44].

### Fluorescence microscopy and image acquisition

Chromosomes were viewed and photographed with a Leica DMLB fluorescence microscope (Leica, Heerbrugg, Switzerland) equipped with a computer-assisted Leica DC 250 digital camera system. Red, green, and blue images were captured in black and white using appropriate filters for TRITC, FITC, and DAPI excitation, respectively. Digital images were pseudo-coloured and combined using IM 1000 Leica software, then imported into Adobe Photoshop, version 7.0 (Adobe, San Jose, California, USA) for final processing.

### HMW DNA isolation

Nuclei were isolated from leaves according to Meyers et al. [31] with some modifications. Fifty grams of young leaves were ground in liquid nitrogen and nuclei were liberated by incubating the cell extract at 4°C for 20 min in HB 1× extraction buffer, plus 0.2% of polyvinylpyrrolidone (PVP 40). PVP was added to the buffer to reduce the production of oxidizing polyphenolic substances. To eliminate cell debris, the leaf homogenate was filtered successively through four layers of cheesecloth then two layers of Miracloth (250 μm), (Calbiochem, UK) and a low speed centrifugation (60 × g for 2 min) was performed. Centrifugation at 850 × g for 8 min at 4°C was followed by a Percoll gradient (37.5%) to separate nuclei from the pectin matrix [45]. The nuclei was washed in 20 mL of HB 1× extraction buffer without β-mercaptoethanol and Triton-100×, and then centrifuged at 850 × g for 8 min at 4°C. Finally the nuclei pellet was resuspended in 1 mL of filtered HB 1× and embedded in 1.2% low-melting-point agarose plugs (InCert Agarose, Cambrex-Bioscience, Rockland, Inc.). An aliquot of the nuclei extraction was evaluated under a microscope using DAPI staining to observe the integrity of the nuclei and the purity of the preparation in terms of organelle contamination. Agarose plugs containing HMW DNA were incubated for 24 h at 50°C in lysis buffer (5% Sodium Lauryl Sarcosyl, 0.625 M EDTA pH 9.0, 50 mg Proteinase K), washed for 1 h at 4°C in inactivation solution (0.5 M EDTA, pH 8.0, 1 mM PMSF), then washed four times for 30 min in TE 10/10 (10 mM Tris-HCl, 10 mM EDTA, pH 8.0). An extra HMW DNA purification step was conducted with a pulsed-field gel electrophoresis (PFGE) using a CHEF Mapper™ XA apparatus (Bio-Rad, U.K.) at 6 V/cm, with 3 s of switch time, and an angle of 120° for 40 min aiming to eliminate the degraded DNA. Agarose plugs were finally washed four times for 30 min in TE 10/1 (10 mM Tris-HCl, 1 mM EDTA, pH 8.0). at 4°C before being used for restriction enzyme digestions.

### BAC library construction

Agarose plugs containing HMW DNA were chopped into small pieces and incubated on ice, with agitation, three times in 1 mL of *Hind*III restriction buffer (Gibco BRL, USA), with buffer exchange every 30 min. Seven units of *Hind*III was added to each chopped plug and allowed to diffuse for 4 hours on ice. For partial digestions, the reactions were incubated for 15 min at 37°C and then stopped by adding one-tenth of the total volume of 0.5 M EDTA, pH 8.0. Partially digested HMW DNA was size-selected by two successive PFGE in 1% GTG SEAKEM agarose gels in 0.5× TBE at 14°C. The first-sizing was performed at 6 V/cm, with 1 s to 50 s of switch-time, and an angle of 120° for 20 h. Two regions from 80 to150 kb and 150 to 250 kb were excised from the gel and loaded onto a new gel. The second-sizing selection was then performed at 6 V/cm, with 3 s of switch time, and an angle of 120° for 20 h. The regions from 100 to 250 kb were cut out from the latter gel and the DNA was recovered through an electro-elution (BIO-RAD/electro-eluter, UK). DNA concentration was estimated in 1% agarose gel in 1× TAE. Several ligation reactions were tested containing different ratios of vector to insert. A constant 30 ng of the commercial vector "pIndigo BAC-5 *HindIII*-Cloning Ready" (Epicentre, USA) was used, and varying amounts of DNA ranging from 50 to 600 ng of HMW DNA were used. One microlitre of ligation was mixed to 20 microlitres of competent *E. coli *cells (ElectroMAX DH10B, Invitrogen) and electro-transformed using a BRL Cell-Porator system according to the manufacturer's recommendations but with a charge rate of 355 volts. Transformants were selected on 2YT plates (tryptone 16 g/L, yeast extract 10 g/L, sodium chloride 5 g/L, agar 16 g/L) containing 12.5 μg/mL of chloramphenicol, 50 μg/mL of 5-bromo-4-chloro-3-indole-β-D-galactopyranoside (X-Gal) and 25 μg/mL of isopropyl-thiogalactoside (IPTG). White colonies were picked using a Q-Pix 2 colony picker robot (Genetix) and transferred to 384-well plates containing 80 μL of 2YT, 7% glycerol. Microplates were incubated for 18–20 h at 37°C and stored at -80°C.

### BAC library screening and DNA isolation

For estimation of BAC clone insert sizes, random individual clones were grown in 100 μL pre-innoculum and then in 3 mL 2YT liquid medium containing chloramphenicol (12.5 μg/mL). BAC DNA was isolated using a QIAGEN BIO-ROBOT 9600 (Qiagen GmbH, Germany). BAC DNA was digested with *Not*I to release the inserts. The digested clones were separated by PFGE at 6 V, a switch time from 5 to 15 s, an angle of 120° and run for 15 h. High-density filters were made using a Q-Pix 2 robot (Genetix). Each high-density filter contained 18,432 double-spotted clones. Hybridisations were performed as described in the Clemson BAC protocols [45]. Filters were exposed for 24 h to Ferrania LifeRay XCG-films.

### Genomic probes

Estimation of organelle contamination in both libraries was evaluated by hybridization of high-density filters using probes from a spinach chloroplast gene, the large Rubisco subunit (1.5 kb) and a wheat mitochondrial gene of cytochrome oxidase *cox I *(1.3 kb) [32]. BAC libraries were also hybridized to probes from single-copy genes that have been defined as legume anchor markers [46] and the *Arachis *resistance gene analogue S1_A_36 [47] (Genbank accession AY157808). This RGA was isolated from the AA genome species *A. stenosperma *and has been found to co-localize with a QTL for resistance to the late-leaf spot *Cercosporidium personatum *[43].

## Authors' contributions

PMG conceived the study, constructed the libraries and drafted the manuscript. KP constructed the libraries and participated in planning the experiments. OG adapted the BAC library construction protocol to Arachis, constructed the libraries, and contributed to the writing of the manuscript. SCLB conceived the study, contributed resources and participated in drafting the manuscript. GS conducted the cytogenetic experiments. CC was responsible for the maintenance of the plants in greenhouse. DJB conceived and coordinated the study and drafted the manuscript. ADH contributed materials and resources, coordinated libraries construction and contributed to the writing of the manuscript. All authors read and approved the manuscript.
